# Therapeutic efficacy of artemether-lumefantrine and molecular markers of antimalarial resistance in Niger, 2022

**DOI:** 10.1186/s12936-025-05679-x

**Published:** 2025-12-29

**Authors:** Ibrahim Maman Laminou, Sanoussi Maman Kabirou, Ibrahima Issa Arzika, Abdou Yahaya, Jehan Ahmed, Awa Bineta Deme, Mamadou Alpha Diallo, Bassirou Ngom, Amy Gaye, Aïta Sene, Djiby Sow, Eric Coulibaly, Zilahatou Bahari-Tohon, Elisha Sanoussi, Daniel Koko, Irene Cavros

**Affiliations:** 1 Niamey-Niger Medical and Health Research Center (CERMES), Niamey, Niger; 2 Republic of Niger National Malaria Control Programme, Niamey, Niger; 3 U.S. President’s Malaria Initiative Impact Malaria Project, Population Services International, Washington, DC USA; 4 International Center for Research and Training in Applied Genomics and Health Surveillance (CIGASS), University Cheikh Anta Diop de Dakar, Dakar, Senegal; 5 U.S. President’s Malaria Initiative, U.S. Agency for International Development, Niamey, Niger; 6 U.S. President’s Malaria Initiative Impact Malaria Project, Population Services International, Niamey, Niger; 7 U.S. President’s Malaria Initiative, U.S. Centers for Disease Control and Prevention, Atlanta, GA USA

**Keywords:** Efficacy, Artemether-lumefantrine, Niger, Antimalarial resistance, Molecular surveillance

## Abstract

**Background:**

From August to October 2022, a therapeutic efficacy study of Niger’s first-line antimalarial, artemether-lumefantrine (AL), was conducted in four sites (Aderbissinat, Boboye, Aguié, and Baban Tabki) to evaluate its therapeutic efficacy and investigate for molecular markers of antimalarial drug resistance.

**Methods:**

Children aged 5 to 15 years old with uncomplicated malaria were assessed in a 28 day in vivo efficacy study. Genotyping using three markers (*msp1, msp2* and the PolyA microsatellite) and match counting using the WHO three-out-of-three algorithm, were used to distinguish recrudescences from new infections. A two-out-of-three algorithm was also utilized as a sensitivity analysis.

**Results:**

PCR uncorrected and corrected efficacy results at day 28 were calculated. Resistance markers were analysed by next-generation sequencing. Uncorrected treatment efficacies were 62.0% (95% CI 54–74) in Aderbissinat, 95.4% (95% CI 91–100) in Aguié, 98.7% (95% CI 96–100) in Boboye, and 50.6% (95% CI 42–62) in Baban Tabki. After PCR correction, AL efficacy was 100%, 97.5%, 100%, and 93.5%, respectively. Marker analysis revealed a high prevalence *of S108N, C59R,* and *N51I* mutations in the *pfdhfr* gene, and *S436A* and *A437G* mutations in the *pfdhps* gene. No validated or candidate *pfkelch13* mutations were observed.

**Conclusion:**

In all four sites evaluated, AL retains therapeutic efficacies above the 90% WHO-recommended threshold using the primary three-out-of-three match criteria. In Aguié and Baban Tabki, efficacy remained above the threshold with certain match criteria and statistical approaches but fell below the cutoff using two-out-of-three matching and per-protocol methods, suggesting emerging efficacy concerns in southern parts of the country.

**Supplementary Information:**

The online version contains supplementary material available at 10.1186/s12936-025-05679-x.

## Background

According to the World Health Organization (WHO), parasite resistance to drugs, vector resistance to insecticides, climate change, humanitarian crises and lack of resources are the main challenges jeopardizing the global fight against malaria [1]. In 2023, the total number of malaria cases worldwide reached 263 million, over 7.9 million of which were in Niger [1]. Niger also ranks third in the world for the highest number of malaria deaths, accounting for 6% of all malaria deaths globally in 2022, behind Nigeria (31%) and the Democratic Republic of the Congo (12%) [2]. Among children under the age of 5 years, malaria prevalence in Niger was found to be 29% in 2021; the incidence of the disease during this same period was 204 cases per 1000 inhabitants [3].

Consequently, malaria poses a significant public health problem in Niger. Efficacious antimalarials help ensure patients recover from malaria and are an essential tool for malaria control. Since it was first introduced in Niger in 2005, artemisinin-based combination therapy (ACT) has been the cornerstone of malaria treatment. Niger lists four artemisinin-based combinations in its national guidelines for the treatment of malaria: artemether-lumefantrine (AL), artesunate-amodiaquine (ASAQ), dihydroartemisinin-piperaquine (DHA-PPQ), and artesunate-pyronaridine (ASPY) [4]. In practice, AL is the primary first-line treatment, as DHA-PPQ and ASPY cost significantly more to procure. ASAQ is also not widely employed as a first-line treatment in Niger because sulfadoxine-pyrimethamine + amodiaquine (SPAQ) is already extensively used in seasonal malaria chemoprevention (SMC)**,** a strategy that provides monthly doses of antimalarials to children under five during the rainy season to prevent malaria. This strategic allocation minimizes the risk of drug resistance to amodiaquine, a critical component of SPAQ, and ensures distinct antimalarials are reserved for prevention versus treatment. However, this also increases drug pressure on AL, Niger’s primary first-line treatment.

Drug resistance is a significant challenge in malaria control, arising when parasites evolve to survive antimalarial treatments, leading to treatment failures and increased disease burden. Resistance can develop due to factors such as widespread drug use, improper dosing, and limited drug diversity, compromising the efficacy of first-line and preventive therapies. To address this, therapeutic efficacy studies (TES) are conducted to monitor the efficacy of antimalarial drugs. These studies help identify emerging resistance, guide treatment policy updates, and ensure that recommended therapies remain effective in controlling malaria and reducing mortality.

The first study on the therapeutic efficacy of AL in Niger was conducted in 2009 at three of the country’s sentinel sites. The corrected efficacy of AL was found to be 96.3% in Tessaoua, 94.1% in Agadez, and 89.6% in Gaya [5]. The most recent study on the therapeutic efficacy of AL was carried out in 2020 and showed efficacy rates of 92.2% in Tessaoua, 97.1% in Agadez, and 98.9% in Gaya [6].

## Methods

### Study design and sites

**Fig. 1:**
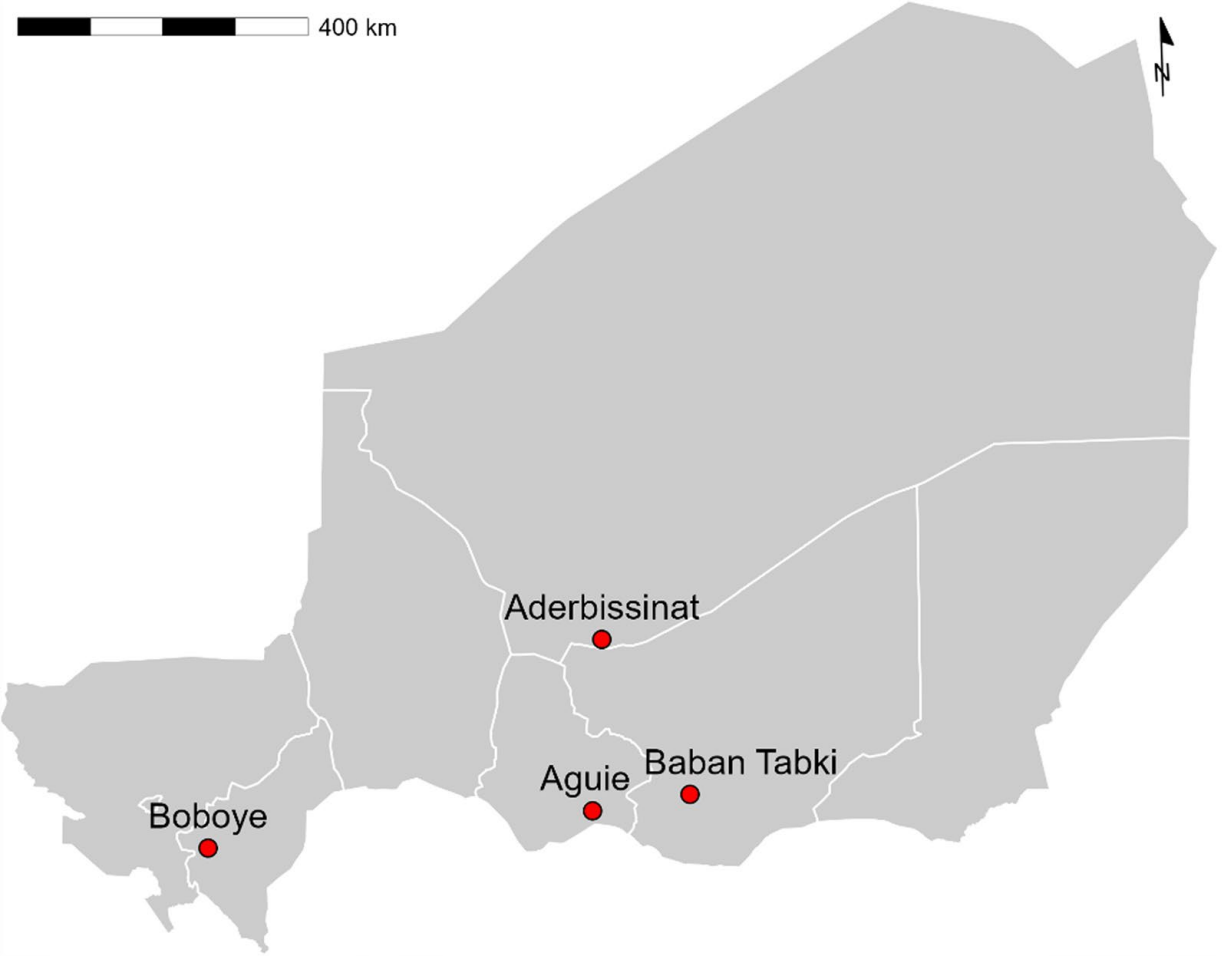
Map of Niger and the four 2022 therapeutic efficacy study sites

This study of AL in patients with uncomplicated *Plasmodium falciparum* malaria was conducted following the standard WHO in vivo protocol for antimalarial efficacy surveillance. It was carried out at four sentinel sites in regions representing the diverse malaria epidemiology in Niger: Centre de Santé Intégré (CSI) of Aderbissinat in the Agadez region, CSI of Baban Tabki in the Zinder region, CSI of Boboye in the Dosso region and CSI of Aguié in the Maradi region (Fig. 1). The study was conducted between August 27 and October 25, 2022, corresponding with Niger’s high malaria transmission season.

### Study population

The study was carried out on patients between six and fifteen years of age with uncomplicated *P. falciparum* malaria who presented at one of the four selected health facilities. Those aged 3 to 59 months were not included to avoid confounding results, as this group is targeted for seasonal malaria chemoprevention using SPAQ.

The study included patients aged between 6 and 15 years, with an axillary temperature equal to or greater than 37.5 °C or a history of fever in the last 24 h, with monospecific *P. falciparum* infection, a parasite density of between 1,000 and 200,000 P/μl, a haemoglobin level greater than 6 g/dl, the ability to take oral medication, and a willingness to comply with the protocol for the duration of the study. The study excluded patients who were malnourished or had a history of antimalarial treatment within the last two weeks, those with general danger signs or severe malaria, those with a mixed malaria infection, or those with a history of hypersensitivity to AL. The inclusion and exclusion criteria for this study are described in further detail in the 2009 WHO protocol for evaluating and monitoring the efficacy of antimalarial drugs for the treatment of uncomplicated *P. falciparum* malaria [7].

### Sample size calculation

A 5% failure rate was assumed at each site based on results from previous studies. With this estimated AL failure rate of 5%, a confidence level of 95%, an estimated precision of 5%, and an estimated loss to follow up of 10%, a minimum of 82 patients was estimated necessary for recruitment at each of the sites, yielding a cumulative total of 328 patients across all four sites.

### Study procedures

Individuals who met the criteria for inclusion in the study were recruited and treated at the sites with AL in accordance with Niger’s malaria national treatment guidelines and then monitored for 28 days. AL was administered twice daily for three days (six doses in total) with dosage determined according to body weight: one tablet for patients 5–14.9 kg, two tablets for patients 15–24.9 kg, three tablets for patients 24–34.9 kg, and four tablets for patients weighing 35 kg or greater. All AL tablets were sourced and pre-approved by the WHO to ensure quality control. The drug was administered with food containing greater than three grams of fat according to manufacturer recommendations. Morning treatments were directly supervised with a minimum post-dose observation period of 30 min at the health facility. Evening doses were administered at home by caregivers; community tracers called caregivers via mobile phone in the evening to ensure no doses were missed. For both morning and evening doses, the full treatment was readministered in cases where vomiting occurred within 30 min of administration. Antipyretics, such as paracetamol, were used to control fever as necessary. Rescue therapy according to national treatment guidelines was also administered in cases of early or late treatment failure [4].

Patients were monitored regularly for 28 days. Follow-up during the 28 day period consisted of a series of fixed-date visits on days 0, 1, 2, 3, 7, 14, 21, and 28 with corresponding clinical examinations and laboratory tests. Additional unscheduled visits occurred if the child was sick or needed clinical care during the follow-up period. Clinical examinations (vital signs and body temperature) were performed at all follow-up visits. Thick and thin Giemsa-stained blood slides were prepared and dried blood spots (DBS) consisting of capillary blood on filter paper were prepared at each visit except for day 1. Due to challenges sourcing the WHO-recommended Whatman 903 (Cytiva^®^) filter papers, Serobuvard (Zoopole^®^) filter papers were utilized.

All participants who had recurrent parasitaemia were managed according to the Niger malaria treatment guidelines and checked to ensure that their malaria infection was cleared.

### Microscopy

Slides were examined by two independent microscopists, and differences in parasite density greater than 50% or differences in species diagnosis were resolved using a third independent microscopist who read the slide and calculated parasite density by averaging the two closest counts. Parasite density, expressed as the number of asexual parasites per µl of blood, was calculated by dividing the number of asexual parasites by the number of white blood cells and then multiplied by an assumed white blood cell density of 6000 per µl. Slides were considered negative if no parasites were seen after examination of 200 oil-immersion fields in a thick blood film.

### Study outcomes

At the end of follow-up, response to treatment was classified according to clinical and parasitological criteria into the WHO-standardized definitions of early therapeutic failure (ETF), late clinical failure (LCF), late parasitological failure (LPF), and adequate clinical and parasitological response (ACPR). ETF was defined as the presence of one of the following: danger signs or severe malaria on day 1, 2 or 3 in the presence of parasitaemia, parasitaemia on day 2 higher than on day 0 irrespective of axillary temperature, parasitaemia on day 3 with axillary temperature ≥ 37.5 °C, and parasitaemia on day 3 ≥ 25% of count on day 0. LCF was defined as the presence of danger signs or severe malaria or axillary temperature ≥ 37.5 °C in the presence of parasitaemia on any day between day 4 and day 28 in patients who did not previously meet any of the criteria of early treatment failure. LPF was defined as the presence of parasitaemia on any day between day 7 and day 28 with axillary temperature < 37.5 °C in patients who did not previously meet any of the criteria of early treatment failure or late clinical failure. ACPR was defined as absence of parasitaemia on day 28, irrespective of axillary temperature, in patients who did not previously meet any of the criteria of early treatment failure, late clinical failure or late parasitological failure.

### Molecular laboratory analysis

For molecular analysis of DBS, parasite DNA was extracted using the Qiagen mini DNA extraction kit (QIAamp^®^). After extraction, the DNA was stored at − 20 °C. Real-time polymerase chain reaction (PET-PCR) was used to confirm the genus and species of the plasmodia. Only mono-specific *P. falciparum* infections were analysed. Samples without amplification (or with Ct > 35) were considered negative.

The genetic polymorphism of the strains was analysed by PCR amplification of the *msp1* and *msp2* genes. Primers specific to different allelic families (K1 and MAD20 for *msp1*, 3D7 and FC27 for *msp2*) and neutral microsatellites (PolyA) were used to distinguish recrudescences from new infections. (7) PCRs for these markers were performed in duplicate on samples taken at day 0 and the day of failure. PCR product sizes for the *msp1* and *msp2* allelic families were determined on a 2% agarose gel under UV light. Any difference in length of 10 bp or less between day 0 and the day of failure for *msp1* and *msp2* was considered a match. PolyA fragment size was determined from capillary electrophoresis using Gene Marker microsatellite analysis software. Any difference in length of 1.5 bp or less between day 0 and the day of failure was considered a match. Interpretation of results was based on 2007 WHO recommendations, using the three-out-of-three match counting algorithm [8]. Recrudescence was classified as when at least one allele at all three markers was common to both the day 0 and day of failure samples. Loci with indeterminate results in either sample were excluded from the denominator, consistent with WHO guidance; thus, outcomes with only one or two successfully typed loci were classified according to the available loci, with recrudescence assigned if all interpretable loci matched and new infection assigned if they did not. The two-out-of-three algorithm was also applied as a sensitivity analysis, but only when all three loci yielded interpretable results (i.e., two matches and one non-match out of three were classified as recrudescence). When one or more loci were indeterminate, both algorithms converged on the same rule: all interpretable loci had to match in order to classify an outcome as recrudescence.

**Fig. 2:**
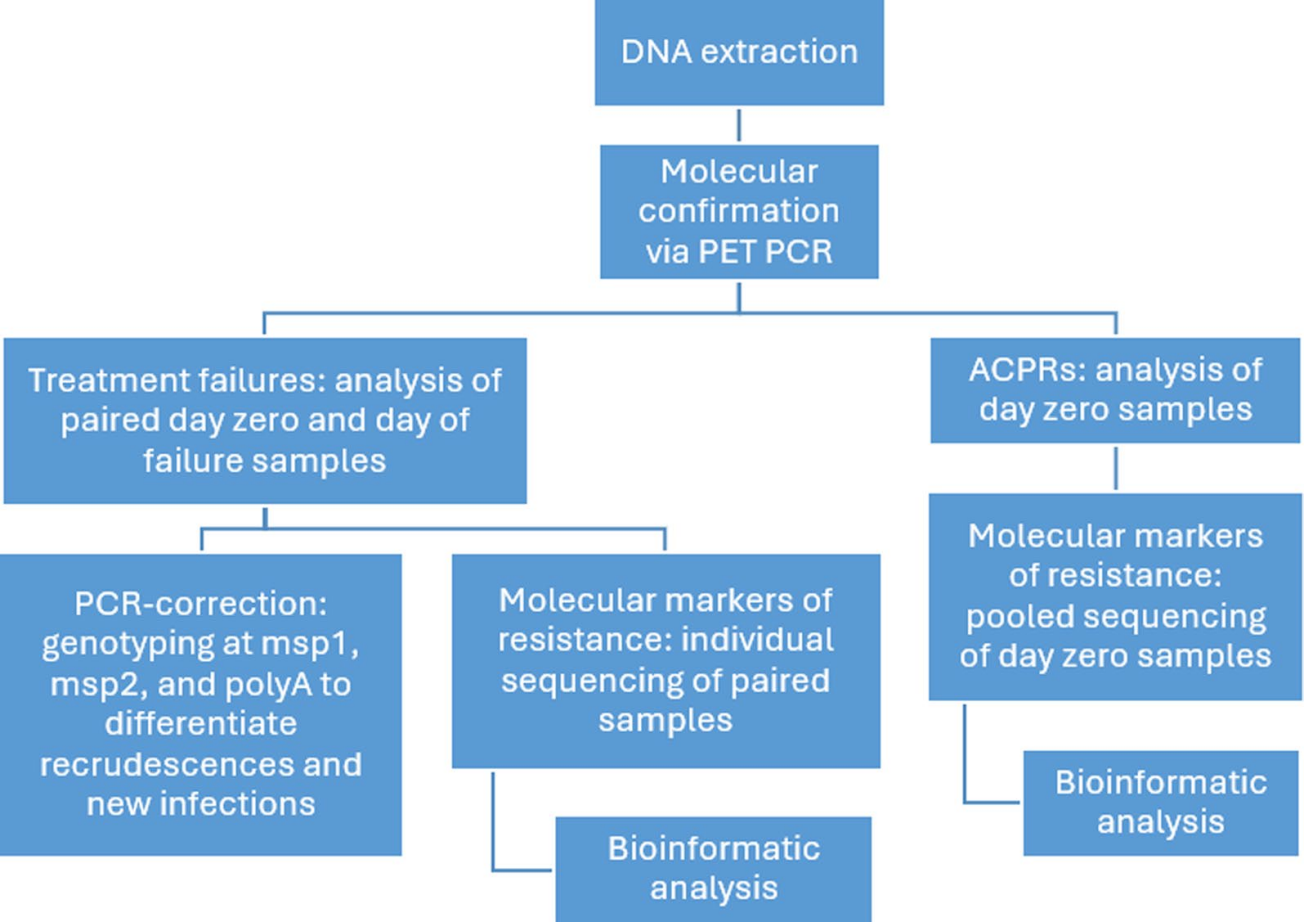
Molecular sample analysis workflow

Five major molecular markers of antimalarial drug resistance were amplified and sequenced: *pfkelch13, pfmdr1, pfcrt, pfdhfr and pfdhps*. This was done using the Malaria Resistance Surveillance, or MaRS, method developed by the CDC, utilizing the Illumina MiSeq platform to sequence amplicons [9]. Figure 2 illustrates the molecular sample analysis workflow, including both genotyping and sequencing processes. All molecular laboratory analyses were performed at the International Center for Research and Training in Applied Genomics and Health Surveillance (CIGASS) in Dakar, Senegal.

### Data management and statistical analysis

Data were collected on a tablet running Kobo Collect software, utilizing double entry to reduce the risk of data entry errors. Data were analysed using R Studio software. Per-protocol uncorrected efficacy was calculated by dividing ACPRs by the number with a primary outcome (i.e., ETF, LCF, LPF, and ACPR). Per protocol PCR-corrected efficacy was calculated by dividing ACPRs by the sum of ACPRs, early treatment failures, and the recrudescent infections; new infections were not included in the numerator or the denominator. Secondary outcomes included uncorrected efficacy and parasite clearance rates measured on day 3.

Kaplan–Meier analysis was used to calculate cumulative drug efficacy at each site, where uncorrected efficacy considered all treatment failures as events and PCR-corrected efficacy considered only recrudescences as events. Those with new infections, protocol violations, or loss to follow-up were excluded from the PCR-corrected per protocol analysis and censored on the day of new infection/protocol violation/loss to follow-up in the Kaplan–Meier analysis.

To assess the presence of molecular markers of antimalarial resistance, sequences were analysed at loci representing the main single nucleotide polymorphisms (SNPs) to be reported for each gene. SNPs and read coverage were identified using Geneious Prime software. For each site, the weighted frequency of variant alleles (VAF) at each polymorphic site was calculated using the following formula:

$$VAF\, = \,\sum {_{i = 1}^{N} \,} VAF_{i} w_{i} /\sum {w_{i} } .$$

### Ethics

This therapeutic efficacy study was authorized by Niger’s National Ethics Committee on 12/07/2022 by letter No. 32/2022/CNERS and was reviewed by the CDC human-subjects office and determined as non-engaged research. All patients included in the study gave their free and informed consent. For minors, informed consent was obtained from parents or legal guardians. Consent forms were dated and signed by both parties. The data collected was anonymous and kept completely confidential.

## Results

### Baseline study population characteristics

Five hundred and twelve (512) patients were screened, including 103 in Aderbissinat, 123 in Aguié, 121 in Baban Tabki and 165 in Boboye. Three hundred and fifty-eight (358) patients were included in the study, including 91 in Aderbissinat, 82 in Aguié, 91 in Baban Tabki and 94 in Boboye. Table 1 shows the characteristics of the study population.

**Table 1:** Baseline characteristics of TES participants by site in Niger, 2022

Artemether-lumefantrine	Study site			
	Aguié (n = 82)	Aderbissinat (n = 91)	Boboye (n = 94)	Baban Tabki (n = 91)
% Female	50%	48%	47%	53%
Median age in years (range)	7 (5–15)	10 (5–15)	8 (5–15)	8 (5–15)
Median weight in kg (range)	20 (11–48)	24 (13–51)	23 (12–56)	21 (13–45)
Mean baseline parasite density in P/µL (range)	49,400 (1000–200,000)	32,970 (1000–188,800)	33,540 (1120–198,400)	44,350 (1200–199,000)
Mean baseline haemoglobin in g/l (range)	10.8 (7–15)	12.0 (6–15)	11.3 (6–16)	11.0 (6–15)

### Response to treatment and tolerance of AL

No treatment-related adverse events were reported during the study at any of the four sites. One hundred and sixty-four paired samples (82 pairs containing one day zero and one day of failure dry blood sample) were genotyped. Table 2 shows the proportions of recrudescences, new infections, failures and adequate clinical and parasitological responses.

**Table 2:** Treatment outcomes and day 3 slide negativity rates by site in Niger, 2022

Artemether-lumefantrine	Study site			
	Aguié (n = 82)	Aderbissinat (n = 91)	Boboye (n = 94)	Baban Tabki (n = 91)
Loss to follow-up or withdrawn	3	4	17	2
Reached study endpoint	79	87	77	89
Day 3 slide negativity rate (95% CI)	100 (94–100)	98 (91–100)	100 (94–100)	100 (95–100)
Early treatment failures	2	0	0	0
Late treatment failures	28	4	1	44
New infections (3/3 match)	25	4	1	38
Recrudescences (3/3 match)	3	0	0	6
Adequate clinical and parasitological response	49	83	76	45

Late treatment failures: late clinical failures + late parasitological failures

PCR: polymerase chain reaction

**Fig. 3:**
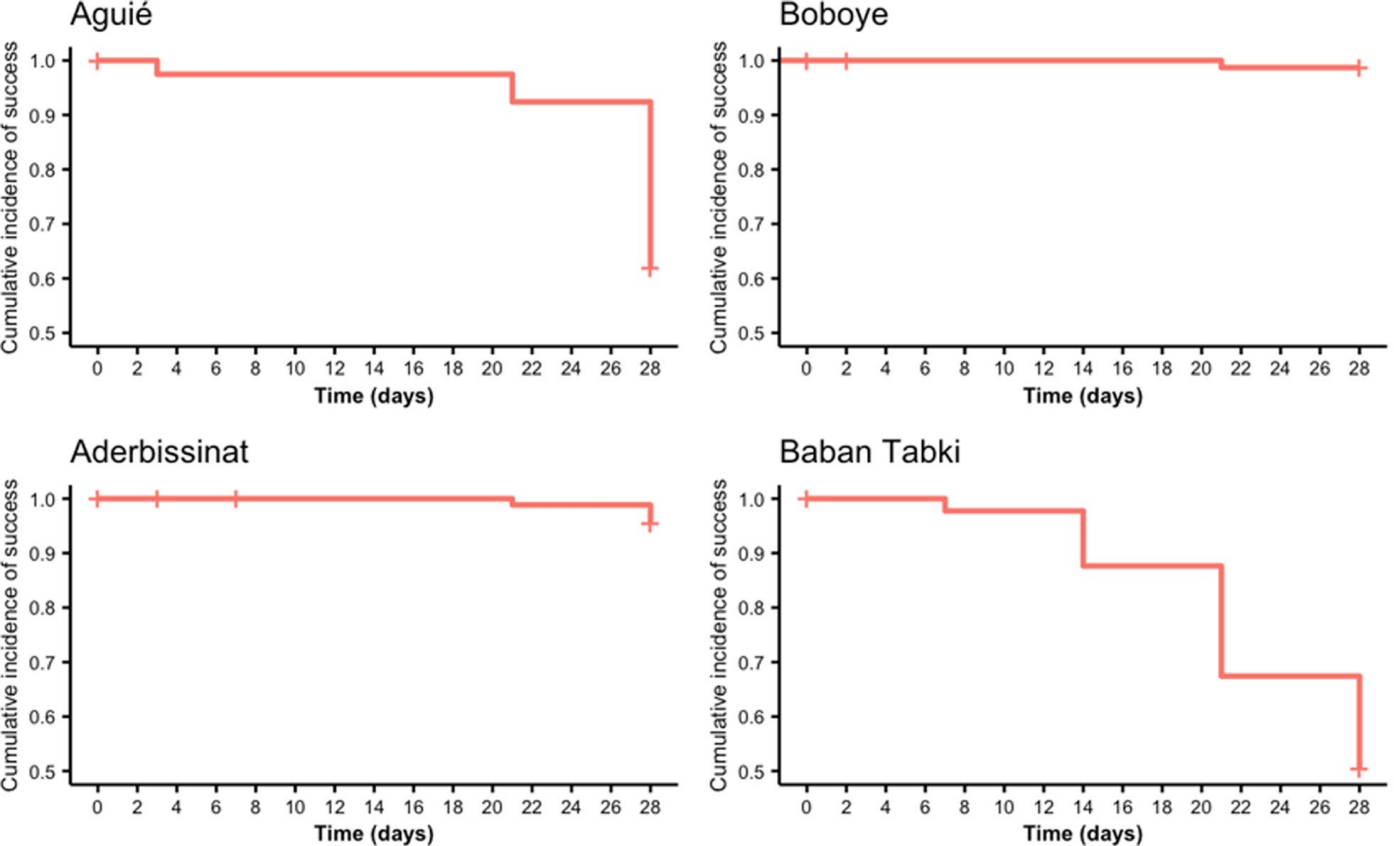
Uncorrected Kaplan–Meier efficacy of artemether-lumefantrine by study site (3/3 match criteria)

**Fig. 4:**
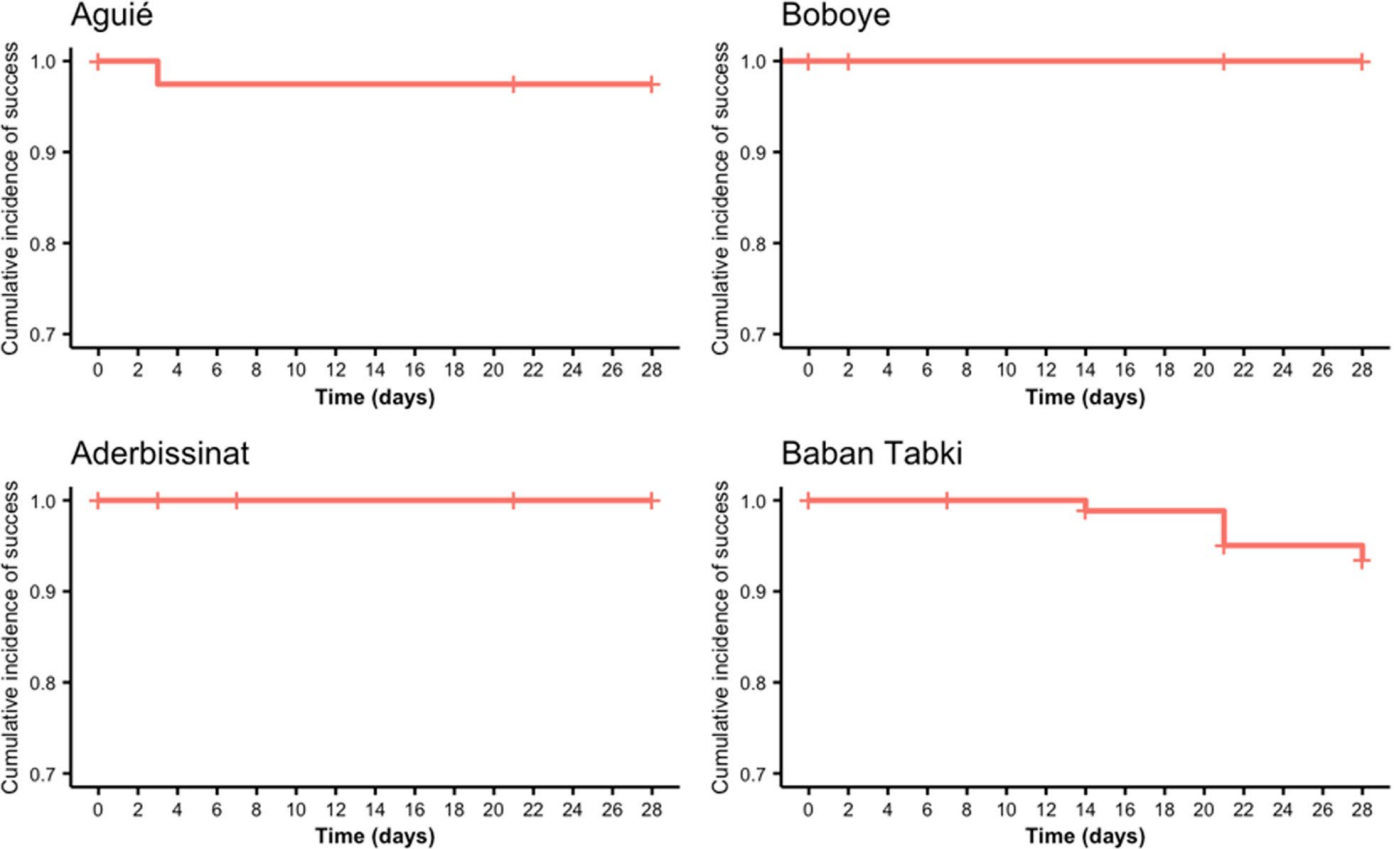
PCR-corrected Kaplan–Meier efficacy of artemether-lumefantrine by study site (3/3 match criteria)

Per protocol and Kaplan–Meier estimates for AL efficacy at each site are found in Table 3 and Figs. 3 and 4 show Kaplan Meier curves before and after PCR correction. Using WHO’s three-out-of-three match algorithm, uncorrected AL efficacy calculated by Kaplan–Meier survival estimation was 62.0% (95% CI 54–74) in Aguié, 95.4% (95% CI 91–100) in Aderbissinat, 98.7% (95% CI 96–100) in Boboye and 50.6% (95% CI 42–62) in Baban Tabki. PCR-corrected Kaplan–Meier AL efficacy, calculated using the recrudescence and new infection classifications from genotyping, was 93.5% (95% CI 88–99) in Aguié, 100% in Aderbissinat, 100% in Boboye, and 92.2% (95% CI 86–98) in Baban Tabki. Notably, using the per protocol method, PCR-corrected efficacy dipped to 90.7% in Aguié and below the WHO’s 90% threshold to 88.2% in Baban Tabki.

**Table 3:** Treatment efficacy among participants reaching study endpoint by study site in Niger, 2022

Artemether-lumefantrine	Study site			
	Aguié (n = 79)	Aderbissinat (n = 87)	Boboye (n = 77)	Baban Tabki (n = 89)
Uncorrected				
Per protocol % (95%CI)	62.0 (51–72)	95.4 (88–98)	98.7 (93–100)	50.6 (40–61)
Kaplan–Meier estimate % (95%CI)	62.0 (54–74)	95.4 (91–100)	98.7 (96–100)	50.6 (42–62)
PCR-corrected				
3/3 match—per protocol % (95%CI)	90.7 (80–96)	100*	100*	88.2 (77–95)
3/3 match—Kaplan–Meier % (95%CI)	93.5 (88–99)	100*	100*	92.2 (86–98)
2/3 match—per protocol % (95%CI)	87.5 (76–94)	100*	98.7 (93–100)	81.8 (70–90)
2/3 match—Kaplan–Meier % (95%CI)	90.8 (85–98)	100*	98.7 (96–100)	87.6 (80–95)

PCR: polymerase chain reaction

^*^Confidence Intervals are undefined

In high transmission settings, such as Niger, it has been suggested that this two-out-of-three approach better balances sensitivity and specificity in addition to better accounting for genetic diversity and complexity of infections in comparison to the three-out-of-three approach [10]. As a sensitivity analysis, PCR-corrected efficacy was also calculated considering samples with matches at two-out-of-three genetic markers as recrudescences, compared to the above three-out-of-three approach. Using the Kaplan–Meier method, PCR-corrected Kaplan–Meier estimates were 90.8% (95% CI 85–98), 100%, 98.7% (95% CI 96–100), and 87.6% (95% CI 80–95) in Aguié, Aderbissinat, Boboye, and Baban Tabki, respectively. Per protocol efficacy using the two-out-of-three approach fell to 87.5% in Aguié, 98.7% in Boboye, and 81.8% in Baban Tabki while remaining steady at 100% in Aderbissinat. A complete genotyping database of treatment failures from all sites can be found in Appendix 1 of the supplemental materials**.**

### Molecular markers of antimalarial resistance

In order to identify any mutations in parasites associated with antimalarial resistance, next generation sequencing (NGS) was utilized at the *pfk13, pfdhfr, pfdhps, pfcrt* and *pfmdr1* genes. All 82 pairs of day zero and day of failure samples were sequenced individually; a total of 18 pools were constructed to analyse the 180 day zero samples from patients classified as ACPRs.

None of the samples analysed had any WHO-validated or candidate *pfk13* mutations associated with artemisinin resistance. At the *pfmdr1* locus, the N86Y mutation was found only in Aguié and Baban Tabki, at rates of 4.5% and 2.4%, respectively. The *mdr1* Y184F mutation thought to be associated with increased resistance to lumefantrine was found in all four sites, ranging from 55.1% in Aguié to 80.2% in Aderbissinat. The resistant *pfcrt* CVIET haplotype, which includes the triple mutant (M74I, N75E, K76T) was absent in Aderbissinat, Aguié, and Boboye and found at a 5% frequency in Baban Tabki.

At the *pfdhfr* gene, the three canonical mutations associated with pyrimethamine resistance (N51I, C59R, S108N) were close to fixation in all sites, ranging from 63–100% in Aderbissinat, 88–95% in Aguié, 95–100% in Boboye, and 89–95% in Baban Tabki. The I164L mutation associated with higher-level pyrimethamine resistance was absent. The *pfdhps* A437G mutation was also widespread, ranging from 67% in Aderbissinat to 96% in Boboye. However, the key *K540E* mutation was not observed at any of the four sites. The critical *pfdhps* A581G mutation was absent in Aderbissinat and Boboye and present at a frequency of 15% in Aguié and 19% in Baban Tabki. In the individual samples for which haplotypes could be inferred, the most common *pfdhfr/pfdhps* haplotype was the canonical IRN/SGKAA quadruple mutant. No canonical quintuple (IRN/SGEAA) mutants were observed at any of the four sites. The *pfdhps* VAGKGS haplotype was observed at a frequency of 8% in Aguié and 22% in Baban Tabki. All mutations observed at the four sites are detailed in Table S2 of the supplemental materials.

### Limitations

The gel electrophoresis methodology used to genotype the *msp1* and *msp2* genes has its limitations compared to the capillary electrophoresis method used for polyA as it offers lower resolution, increasing the risk of misidentifying DNA fragments with similar band lengths, which could lead to ambiguous or incorrect genotype calls. Gel electrophoresis also has lower sensitivity, which has a higher risk of failure to detect minor alleles particularly in cases with mixed infections.

Evening doses of AL were also not directly observed; although study staff placed evening reminder calls to caregivers, the lack of direct observation makes it difficult to rule out suboptimal adherence as a contributing factor to the lower-than-expected efficacy observed in some sites.

## Discussion

Overall, the results of this study align with prior evaluations by Niger’s National Malaria Control Programme and other studies in West Africa. However, for the first time, treatment efficacy of AL fell below the 90% WHO threshold for considering alternative treatment in two sites (Aguié and Baban Tabki) using the two-out-of-three match criteria and per protocol PCR-corrected efficacy; using the two-out-of-three match criteria and Kaplan–Meier PCR-corrected efficacy, one site (Baban Tabki) fell below 90%. In Aguié, PCR-corrected efficacy ranged from 87.5% to 93.5% depending on the statistical method and match criteria used; PCR-corrected efficacy in Baban Tabki had an even wider range between 81.8% and 92.2%. In Aderbissinat and Boboye, efficacy remained consistently high regardless of method. Given the limitations with the gel electrophoresis genotyping methodology—which may reduce confidence in distinguishing recrudescences from new infections—the low uncorrected efficacy estimates (62.0% in Aguié and 50.6% in Baban Tabki) warrant attention, as they may offer a complementary perspective on treatment performance in this high-transmission setting.

Low AL efficacy below or near the 90% WHO threshold has also been observed in nearby countries, such as Burkina Faso in 2021 [11] and Mali in 2016 [12]. Until quite recently, numerous studies from across Africa showed a low prevalence of *pfk13* propeller domain mutations and a very low prevalence of mutations previously associated with resistance in Southeast Asia [13, 14]. The first instances of confirmed artemisinin partial resistance on the continent have emerged in East Africa [15–24]; spread to nearby countries is imminent without close monitoring and ACT diversification.

Molecular sequencing revealed no WHO-validated *pfk13* mutations linked to artemisinin resistance; this finding is supported by other recent molecular studies in Niger [25, 26]. For *pfcrt*, the resistant CVIET haplotype was rare, suggesting that chloroquine resistance remains limited to isolated pockets. Wild-type N86 alleles have re-emerged as predominant, likely due to diminished chloroquine use. The *pfmdr1* gene analysis showed high prevalence of the Y184F mutation (55%−80%), associated with reduced lumefantrine sensitivity. This and the absence of the haplotype containing the N86Y, Y184F, and D1246Y alleles suggests potential reduced susceptibility to lumefantrine, which is in alignment with prior findings in Niger [27]. However, mutations in *pfdhfr* and *pfdhps* indicated widespread SP resistance, with the *pfdhfr* triple mutant (N51I, C59R, S108N) highly prevalent and the *pfdhps* A437G mutation present at high frequencies (67%−96%). The critical *pfdhps* A581G mutation, associated with high-level SP resistance, was absent in Aderbissinat and Boboye but observed at moderate levels in Aguié (15%) and Baban Tabki (19%), suggesting emerging resistance in southern Niger potentially driven by selective drug pressure. Combined findings for *pfdhfr* and *pfdhps* highlight a geographically localized emergence of SP-resistant strains that have not yet spread to western sites such as Aderbissinat and Boboye.

These findings have implications for IPTp and SMC programs which rely on SP. While SP remains effective in western Niger, moderate levels of *pfdhps* A581G in southern Niger suggest that IPTp and SMC efficacy could decline if resistance spreads further. These results highlight the need for ongoing molecular surveillance and expanded monitoring to ensure that these interventions remain viable.

## Conclusion

This study confirms that AL remains effective for treating uncomplicated malaria in Niger in the areas of Aderbissinat and Boboye where efficacy consistently exceeded the WHO threshold of 90% across all analytic methods. While in Aguié and Baban Tabki efficacy remained above the threshold with certain match criteria and statistical approaches, lower estimates using two-out-of-three matching and per-protocol methods suggest that concerns may be emerging. It is worth considering the diversification of first-line ACT in these southern regions to reduce selective pressure on AL and preserve its long-term efficacy.

Molecular analysis revealed no mutations associated with artemisinin resistance, and low prevalence of *pfcrt* mutations linked to chloroquine resistance, suggesting these mechanisms are not widespread. The *pfdhps* A581G mutation associated with reduced SP efficacy was detected at moderate levels in southern Niger—including Aguié and Baban Tabki—further raising concern about localized resistance and potentially limiting the effectiveness of SP-based interventions such as SMC and IPTp. The high prevalence of *pfmdr1* Y184F suggests reduced lumefantrine sensitivity, though the absence of triple mutant haplotypes indicates that widespread multidrug resistance has not yet emerged. Overall, these findings support continued use of AL as a first-line treatment in some areas, while underscoring the need for targeted molecular surveillance—particularly in southern Niger—to monitor evolving resistance patterns.

## Disclaimer

The findings and conclusions in this report are those of the authors and do not necessarily represent the official position of the U.S. Centers for Disease Control and Prevention or the U.S. President’s Malaria Initiative.

## Supplementary Information

Supplementary Material 1

## Data Availability

Data is provided within the supplementary information files.
