# Supplementary material for: Therapeutic efficacy of artemether-lumefantrine and molecular markers of antimalarial resistance in Niger, 2022
Source: Malar J. 2025 Dec 29;24:448. doi: 10.1186/s12936-025-05679-x (PMC12752158; doi:10.1186/s12936-025-05679-x)
Supplement: Supplementary file 1 — Supplementary Material 1 [file 12936_2025_5679_MOESM1_ESM.docx]

Table S1. Allele-level genotyping data supporting PCR-corrected classification of treatment outcomes

| **Sample.ID** | **K1_1** | **K1_2** | **MAD20_1** | **RO33_1** | **3D7_1** | **3D7_2** | **FC27_1** | **FC27_2** | **polya_1** | **polya_2** | **polya_3** | **msp1** | **msp2** | **Poly-a** | **>=2/3** | **=3/3** |
| --- | --- | --- | --- | --- | --- | --- | --- | --- | --- | --- | --- | --- | --- | --- | --- | --- |
| AG4D0 | 220 |  |  |  |  |  | 350 |  | 168 |  |  | **NI** | **NI** | **R** | **NI** | **NI** |
| AG4D28 | 280 |  |  |  | 550 |  | 500 |  | 150 | 156 | 168 |  |  |  |  |  |
| AG10D0 | 210 |  | 240 |  |  |  | 350 | 500 | 144 | 171 |  | **IND** | **IND** | **NI** | **NI** | **NI** |
| AG10D28 |  |  |  |  |  |  |  |  | 159 |  |  |  |  |  |  |  |
| AG13D0 | 220 |  |  |  | 600 |  |  |  | 153 |  |  | **NI** | **IND** | **NI** | **NI** | **NI** |
| AG13D28 |  |  |  | 150 |  |  |  |  | 168 |  |  |  |  |  |  |  |
| AG22D0 | 250 |  |  |  | 600 |  | 300 |  | 150 |  |  | **NI** | **IND** | **NI** | **NI** | **NI** |
| AG22D28 | 180 |  | 240 | 150 |  |  |  |  | 159 | 165 |  |  |  |  |  |  |
| AG26D0 | 200 |  |  |  | 600 |  |  |  | 150 |  |  | **NI** | **NI** | **NI** | **NI** | **NI** |
| AG26D28 | 160 | 220 |  |  | 550 | 650 |  |  | 144 | 147 |  |  |  |  |  |  |
| AG29D0 | 250 |  |  |  | 650 |  |  |  | 153 | 159 |  | **R** | **R** | **NI** | **R** | **NI** |
| AG29D28 | 250 |  |  | 150 | 650 |  |  |  | 156 |  |  |  |  |  |  |  |
| AG38D0 |  |  |  | 150 | 700 |  |  |  | 153 | 168 |  | **NI** | **IND** | **NI** | **NI** | **NI** |
| AG38D21 |  |  | 250 |  |  |  |  |  | 144 |  |  |  |  |  |  |  |
| AG39D0 |  |  | 250 |  | 550 |  |  |  | 156 |  |  | **NI** | **NI** | **R** | **NI** | **NI** |
| AG39D28 | 260 |  | 180 |  | 600 |  |  |  | 156 |  |  |  |  |  |  |  |
| AG41D0 | 250 |  |  |  |  |  | 350 |  | 147 |  |  | **NI** | **NI** | **NI** | **NI** | **NI** |
| AG41D28 | 160 |  |  |  | 600 |  |  |  | 153 |  |  |  |  |  |  |  |
| AG42D0 | 250 |  |  |  |  |  | 400 |  | 159 |  |  | **NI** | **NI** | **NI** | **NI** | **NI** |
| AG42D28 | 200 |  |  |  |  |  | 350 |  | 165 |  |  |  |  |  |  |  |
| AG43D0 | 260 |  |  |  | 500 |  |  |  | 171 | 180 |  | **NI** | **NI** | **NI** | **NI** | **NI** |
| AG43D28 | 210 |  |  |  |  |  | 400 |  | 162 | 168 | 189 |  |  |  |  |  |
| AG44D0 |  |  |  |  |  |  |  |  | 138 | 156 |  | **IND** | **IND** | **NI** | **NI** | **NI** |
| AG44D28 | 180 | 200 | 250 |  | 550 |  |  |  | 153 | 165 | 171 |  |  |  |  |  |
| AG47D0 |  |  |  |  |  |  |  |  |  |  |  | **IND** | **IND** | **IND** | **IND** | **IND** |
| AG47D28 | 200 |  |  |  | 400 |  |  |  | 150 |  |  |  |  |  |  |  |
| AG48D0 | 200 |  | 260 |  | 750 |  | 350 |  | 156 | 186 |  | **IND** | **IND** | **IND** | **IND** | **IND** |
| AG48D28 |  |  |  |  |  |  |  |  |  |  |  |  |  |  |  |  |
| AG55D0 | 160 |  | 300 | 150 |  |  |  |  | 174 |  |  | **NI** | **IND** | **NI** | **NI** | **NI** |
| AG55D28 | 140 | 200 | 280 |  | 600 |  | 400 |  | 162 | 165 |  |  |  |  |  |  |
| AG56D0 | 180 |  | 260 | 150 | 800 | 500 |  |  | 156 |  |  | **NI** | **R** | **NI** | **NI** | **NI** |
| AG56D28 | 200 | 120 |  |  | 500 |  | 350 |  | 147 | 150 |  |  |  |  |  |  |
| AG62D0 | 200 |  |  |  | 550 |  |  |  | 159 |  |  | **IND** | **IND** | **IND** | **IND** | **IND** |
| AG62D28 |  |  |  |  |  |  |  |  |  |  |  |  |  |  |  |  |
| AG123D0 |  |  | 250 |  |  |  |  |  | 156 |  |  | **NI** | **IND** | **R** | **NI** | **NI** |
| AG123D28 | 180 |  |  |  |  |  |  |  | 156 |  |  |  |  |  |  |  |
| AG119D0 | 200 |  |  |  | 600 |  |  |  | 159 |  |  | **IND** | **IND** | **IND** | **IND** | **IND** |
| AG119D21 |  |  |  |  |  |  |  |  |  |  |  |  |  |  |  |  |
| AG117D0 | 250 |  |  |  | 500 |  |  |  | 135 |  |  | **IND** | **NI** | **NI** | **NI** | **NI** |
| AG117D28 |  |  |  |  | 550 |  |  |  | 162 |  |  |  |  |  |  |  |
| AG114D0 |  |  | 200 |  |  |  |  |  | 147 | 150 | 162 | **NI** | **IND** | **NI** | **NI** | **NI** |
| AG114D28 | 200 |  |  |  |  |  |  |  | 156 |  |  |  |  |  |  |  |
| AG110D0 | 210 |  | 180 | 150 |  |  |  |  | 153 | 159 |  | **NI** | **IND** | **NI** | **NI** | **NI** |
| AG110D28 | 250 |  |  |  | 400 |  |  |  | 150 |  |  |  |  |  |  |  |
| AG107D0 |  |  |  |  |  |  |  |  |  |  |  | **IND** | **IND** | **IND** | **IND** | **IND** |
| AG107D21 |  |  |  |  |  |  |  |  |  |  |  |  |  |  |  |  |
| AG103D0 |  |  |  |  |  |  |  |  |  |  |  | **IND** | **IND** | **IND** | **IND** | **IND** |
| AG103D28 |  |  |  |  |  |  |  |  |  |  |  |  |  |  |  |  |
| AG102D0 | 210 | 300 |  |  | 700 |  | 450 |  | 153 | 165 |  | **NI** | **R** | **R** | **R** | **NI** |
| AG102D21 | 250 |  |  |  | 700 |  |  |  | 153 |  |  |  |  |  |  |  |
| AG59D0 | 300 |  |  |  |  |  | 350 |  | 162 |  |  | **IND** | **IND** | **NI** | **NI** | **NI** |
| AG59D28 |  |  |  |  |  |  |  |  | 150 |  |  |  |  |  |  |  |
| AG61D0 | 200 |  | 220 |  | 400 |  | 300 |  | 162 | 177 |  | **R** | **IND** | **NI** | **NI** | **NI** |
| AG61D28 | 210 | 300 |  |  |  |  |  |  | 147 | 153 |  |  |  |  |  |  |
| AG69D0 |  |  | 200 | 150 | 500 | 400 |  |  | 135 |  |  | **R** | **NI** | **NI** | **NI** | **NI** |
| AG69D28 | 250 |  | 210 | 150 | 450 |  | 300 |  | 165 |  |  |  |  |  |  |  |
| AG72D0 | 200 |  |  |  |  |  |  |  | 171 |  |  | **NI** | **IND** | **NI** | **NI** | **NI** |
| AG72D28 | 350 |  |  |  | 450 |  | 300 |  | 150 |  |  |  |  |  |  |  |
| AG81D0 |  |  |  | 150 |  |  |  |  | 147 |  |  | **NI** | **IND** | **NI** | **NI** | **NI** |
| AG81D28 |  |  | 250 |  |  |  |  |  | 141 |  |  |  |  |  |  |  |
| AG82D0 | 200 |  |  |  |  |  | 300 |  | 156 | 159 |  | **NI** | **NI** | **NI** | **NI** | **NI** |
| AG82D28 | 350 |  |  |  | 350 |  |  |  | 153 |  |  |  |  |  |  |  |
| AG88D0 |  |  |  |  |  |  |  |  | 150 |  |  | **IND** | **IND** | **NI** | **NI** | **NI** |
| AG88D21 |  |  |  |  |  |  |  |  | 147 | 186 | 195 |  |  |  |  |  |
| AG92D0 | 250 |  |  |  | 400 |  | 500 |  | 147 |  |  | **NI** | **IND** | **NI** | **NI** | **NI** |
| AG92D28 |  |  |  | 150 |  |  |  |  | 165 |  |  |  |  |  |  |  |
| BT1D0 | 250 |  |  |  |  |  |  |  | 147 | 153 |  | **IND** | **IND** | **NI** | **NI** | **NI** |
| BT1D21 |  |  |  |  |  |  |  |  | 159 |  |  |  |  |  |  |  |
| BT6D0 |  |  |  |  |  |  |  |  | 159 |  |  | **IND** | **IND** | **R** | **R** | **R** |
| BT6D21 |  |  |  |  |  |  |  |  | 159 |  |  |  |  |  |  |  |
| BT9D0 | 280 |  |  |  |  |  | 350 | 400 | 168 |  |  | **IND** | **R** | **IND** | **R** | **R** |
| BT9D28 |  |  |  |  |  |  | 350 | 400 |  |  |  |  |  |  |  |  |
| BT13D0 |  |  |  |  |  |  |  |  | 153 |  |  | **IND** | **IND** | **R** | **R** | **R** |
| BT13D21 | 180 | 300 | 150 |  | 500 | 600 |  |  | 144 | 153 |  |  |  |  |  |  |
| BT14D0 | 200 |  | 200 |  |  |  |  |  | 153 |  |  | **NI** | **IND** | **R** | **NI** | **NI** |
| BT14D28 | 180 |  |  |  | 500 |  |  |  | 153 |  |  |  |  |  |  |  |
| BT19D0 |  |  |  |  |  |  |  |  | 153 |  |  | **IND** | **IND** | **R** | **R** | **R** |
| BT19D28 | 250 |  |  |  |  |  | 350 |  | 153 |  |  |  |  |  |  |  |
| BT21D0 | 280 |  | 200 | 150 | 550 |  | 500 |  | 174 |  |  | **R** | **IND** | **NI** | **NI** | **NI** |
| BT21D28 | 280 |  |  |  |  |  |  |  | 153 | 165 |  |  |  |  |  |  |
| BT31D0 | 250 |  | 210 | 150 | 500 | 700 |  |  | 159 | 180 |  | **R** | **IND** | **IND** | **R** | **R** |
| BT31D14 |  |  | 200 | 150 |  |  |  |  |  |  |  |  |  |  |  |  |
| BT25D0 | 250 |  | 200 |  | 500 |  | 400 |  | 156 |  |  | **R** | **NI** | **R** | **R** | **NI** |
| BT25D21 | 250 |  | 200 | 150 | 450 |  |  |  | 156 |  |  |  |  |  |  |  |
| BT29D0 | 250 |  | 210 |  | 450 |  |  |  | 153 | 156 | 174 | **NI** | **IND** | **R** | **NI** | **NI** |
| BT29D21 | 300 |  |  | 150 |  |  |  |  | 153 | 156 | 159 |  |  |  |  |  |
| BT32 D0 |  |  | 200 |  | 550 |  |  |  | 168 |  |  | **NI** | **NI** | **NI** | **NI** | **NI** |
| BT32 D28 |  |  |  | 150 | 700 |  |  |  | 156 |  |  |  |  |  |  |  |
| BT37D0 | 300 |  |  |  |  |  |  |  | 147 | 162 |  | **NI** | **IND** | **NI** | **NI** | **NI** |
| BT37D14 | 350 |  |  |  | 550 |  |  |  | 156 |  |  |  |  |  |  |  |
| BT46D0 |  |  |  | 150 | 500 |  |  |  | 153 |  |  | **R** | **R** | **NI** | **R** | **NI** |
| BT46D28 | 250 |  |  | 150 | 500 |  | 300 |  | 144 | 171 |  |  |  |  |  |  |
| BT51D0 |  |  | 200 |  | 600 |  | 400 | 700 | 165 |  |  | **R** | **NI** | **NI** | **NI** | **NI** |
| BT51D21 | 250 |  | 200 |  | 500 |  | 350 |  | 159 |  |  |  |  |  |  |  |
| BT52D0 | 220 |  |  |  |  |  | 400 |  | 174 |  |  | **IND** | **IND** | **NI** | **NI** | **NI** |
| BT52D28 |  |  |  |  |  |  |  |  | 159 | 162 |  |  |  |  |  |  |
| BT53D0 |  |  |  |  |  |  |  |  | 153 |  |  | **IND** | **IND** | **NI** | **NI** | **NI** |
| BT53D14 |  |  |  |  |  |  |  |  | 177 |  |  |  |  |  |  |  |
| BT56D0 | 350 |  |  | 150 | 600 |  | 600 |  |  |  |  | **NI** | **IND** | **IND** | **NI** | **NI** |
| BT56D14 |  |  | 200 |  |  |  |  |  | 156 |  |  |  |  |  |  |  |
| BT57D0 | 300 |  |  |  | 550 |  |  |  | 150 |  |  | **NI** | **NI** | **IND** | **NI** | **NI** |
| BT57D14 | 400 |  |  |  | 800 |  |  |  |  |  |  |  |  |  |  |  |
| BT60D0 | 300 |  |  |  |  |  | 350 |  | 153 |  |  | **NI** | **NI** | **NI** | **NI** | **NI** |
| BT60D21 | 200 |  | 150 |  | 500 |  | 300 |  | 168 |  |  |  |  |  |  |  |
| BT63D0 | 250 |  |  |  | 600 |  |  |  | 171 |  |  | **NI** | **NI** | **NI** | **NI** | **NI** |
| BT63D28 |  |  | 200 | 110 | 550 |  |  |  | 159 |  |  |  |  |  |  |  |
| BT68D0 | 300 |  | 200 |  | 600 |  |  |  | 159 |  |  | **NI** | **NI** | **NI** | **NI** | **NI** |
| BT68D21 | 250 |  |  |  |  |  | 350 | 500 | 153 | 156 |  |  |  |  |  |  |
| BT69D0 | 180 | 200 | 200 | 110 |  |  |  |  | 150 | 156 |  | **R** | **IND** | **R** | **R** | **R** |
| BT69D21 |  |  | 200 | 110 |  |  |  |  | 150 | 156 |  |  |  |  |  |  |
| BT72D0 | 280 |  |  |  |  |  |  |  | 156 |  |  | **NI** | **IND** | **NI** | **NI** | **NI** |
| BT72D28 | 250 |  |  | 150 | 500 |  | 450 |  | 159 |  |  |  |  |  |  |  |
| BT74D0 |  |  | 220 |  |  |  |  |  | 156 |  |  | **NI** | **IND** | **NI** | **NI** | **NI** |
| BT74D7 | 250 |  |  |  | 600 |  |  |  | 132 |  |  |  |  |  |  |  |
| BT75D0 | 280 |  | 250 | 150 | 600 |  |  |  | 144 | 150 |  | **IND** | **IND** | **NI** | **NI** | **NI** |
| BT75D28 |  |  |  |  |  |  |  |  | 147 | 162 |  |  |  |  |  |  |
| BT85D0 |  |  |  |  |  |  |  |  | 159 |  |  | **IND** | **IND** | **NI** | **NI** | **NI** |
| BT85D21 |  |  | 250 |  | 600 |  |  |  | 156 | 162 |  |  |  |  |  |  |
| BT86D0 |  |  | 250 | 150 | 550 |  |  |  | 153 |  |  | **NI** | **NI** | **NI** | **NI** | **NI** |
| BT86D28 | 280 |  | 200 |  | 500 |  |  |  | 159 |  |  |  |  |  |  |  |
| BT88D0 | 250 |  |  | 150 | 600 |  | 350 | 550 | 153 |  |  | **R** | **NI** | **R** | **R** | **NI** |
| BT88D28 | 250 |  |  |  | 800 |  |  |  | 153 |  |  |  |  |  |  |  |
| BT89D0 |  |  | 250 |  | 500 |  |  |  | 159 | 174 |  | **IND** | **IND** | **NI** | **NI** | **NI** |
| BT89D21 |  |  |  |  |  |  |  |  | 147 |  |  |  |  |  |  |  |
| BT91D0 |  |  | 220 |  |  |  |  |  | 153 | 156 |  | **NI** | **IND** | **NI** | **NI** | **NI** |
| BT91D14 | 280 |  |  |  |  |  |  |  | 171 |  |  |  |  |  |  |  |
| BT93D0 | 250 |  |  |  |  |  |  |  | 153 |  |  | **NI** | **IND** | **NI** | **NI** | **NI** |
| BT93D28 | 300 |  |  |  |  |  |  |  | 168 |  |  |  |  |  |  |  |
| BT94D0 | 300 |  |  |  | 500 |  |  |  | 150 |  |  | **R** | **R** | **NI** | **R** | **NI** |
| BT94D28 | 300 |  |  | 150 | 500 |  | 500 |  | 159 |  |  |  |  |  |  |  |
| BT95D0 | 280 |  |  |  | 500 |  |  |  | 171 | 189 |  | **NI** | **NI** | **NI** | **NI** | **NI** |
| BT95D28 |  |  |  | 150 | 600 |  |  |  | 159 |  |  |  |  |  |  |  |
| BT96D0 | 300 |  | 210 |  | 800 |  |  |  | 183 |  |  | **R** | **NI** | **IND** | **NI** | **NI** |
| BT96D28 |  |  | 200 |  | 600 |  | 400 |  |  |  |  |  |  |  |  |  |
| BT98D0 | 250 |  | 300 |  |  |  | 350 |  | 153 | 168 |  | **NI** | **NI** | **NI** | **NI** | **NI** |
| BT98D21 | 280 |  |  | 150 | 500 | 800 | 400 | 700 | 138 |  |  |  |  |  |  |  |
| BT100D0 | 250 | 450 | 200 | 200 | 500 |  | 300 |  | 168 |  |  | **R** | **IND** | **NI** | **NI** | **NI** |
| BT100D14 | 250 |  |  |  |  |  |  |  | 153 |  |  |  |  |  |  |  |
| BT101D0 |  |  | 200 | 150 |  |  |  |  | 153 |  |  | **R** | **IND** | **NI** | **NI** | **NI** |
| BT101D21 | 350 |  |  | 150 | 600 |  |  |  | 162 |  |  |  |  |  |  |  |
| BT104D0 | 320 |  |  |  | 500 |  |  |  | 153 |  |  | **R** | **IND** | **NI** | **NI** | **NI** |
| BT104D21 | 320 |  | 210 |  |  |  |  |  | 150 |  |  |  |  |  |  |  |
| BT107D0 |  |  | 200 |  |  |  |  |  | 147 | 156 |  | **R** | **IND** | **NI** | **NI** | **NI** |
| BT107D21 | 400 |  | 200 | 150 | 550 |  |  |  | 171 |  |  |  |  |  |  |  |
| BT109D0 |  |  |  |  | 500 |  |  |  | 159 |  |  | **IND** | **R** | **NI** | **NI** | **NI** |
| BT109D21 | 380 | 300 | 210 |  | 500 |  | 350 |  | 144 | 150 |  |  |  |  |  |  |
| BT117D0 | 350 |  |  |  | 550 |  |  |  | 153 |  |  | **NI** | **NI** | **NI** | **NI** | **NI** |
| BT117D28 | 320 |  |  | 150 | 600 |  |  |  | 144 |  |  |  |  |  |  |  |
| BT114D0 | 380 |  |  |  |  |  |  |  | 159 | 165 |  | **NI** | **IND** | **R** | **NI** | **NI** |
| BT114D14 | 350 |  |  | 150 |  |  | 400 |  | 165 |  |  |  |  |  |  |  |
| BT113D0 | 350 |  |  | 150 | 550 |  | 300 |  | 153 |  |  | **NI** | **IND** | **NI** | **NI** | **NI** |
| BT113D28 |  |  | 200 |  |  |  |  |  | 150 |  |  |  |  |  |  |  |
| BT116D0 | 380 |  |  |  | 600 |  |  |  | 162 |  |  | **NI** | **NI** | **NI** | **NI** | **NI** |
| BT116D21 | 250 |  |  |  | 500 |  |  |  | 165 |  |  |  |  |  |  |  |
| ADT43D0 |  |  |  |  |  |  |  |  | 174 |  |  | **IND** | **IND** | **NI** | **NI** | **NI** |
| ADT43D28 |  |  |  |  |  |  |  |  | 132 | 156 |  |  |  |  |  |  |
| ADT103D0 |  |  |  |  |  |  | 400 |  | 147 |  |  | **IND** | **IND** | **NI** | **NI** | **NI** |
| ADT103D28 |  |  |  | 150 |  |  |  |  | 156 | 159 |  |  |  |  |  |  |
| ADT102D0 | 250 |  |  |  | 500 |  |  |  | 168 |  |  | **IND** | **IND** | **NI** | **NI** | **NI** |
| ADT102D28 |  |  |  |  |  |  |  |  | 153 |  |  |  |  |  |  |  |
| ADT11D0 | 250 |  |  |  |  |  |  |  | 153 | 156 | 165 | **NI** | **IND** | **NI** | **NI** | **NI** |
| ADT11D28 | 280 |  | 200 |  | 500 |  | 300 |  | 168 |  |  |  |  |  |  |  |
| BO29D0 | 200 |  |  |  | 500 |  |  |  | 144 |  |  | **R** | **R** | **NI** | **R** | **NI** |
| BO29D21 | 200 |  |  |  | 500 |  |  |  | 171 |  |  |  |  |  |  |  |

NI: New Infection; R: Recrudescence; IND: Indeterminate

Bin sizes: msp1 (10bp); msp2 (10bp); polya (1.5bp)

Indeterminate loci were excluded from the denominator in both the 3/3 and 2/3 algorithms; the 2/3 rule was only applied when all three loci had interpretable results.

Table S2. Mutations and haplotypes observed by site in Niger’s 2022 TES

| WHO mutation classification | Mutation | Aderbissinat | Aguié | Boboye | Baban Tabki |
| --- | --- | --- | --- | --- | --- |
| ***Pfk13*** | | | | | |
| Validated | C580Y | 0.0% (n=14) | 0.0% (n=91) | 0.0% (n=2) | 0.0% (n=109) |
| Validated | R539T | 0.0% (n=14) | 0.0% (n=91) | 0.0% (n=2) | 0.0% (n=110) |
| Validated | Y493H | 0.0% (n=14) | 0.0% (n=91) | 0.0% (n=2) | 0.0% (n=107) |
| Validated | F446I | 0.0% (n=14) | 0.0% (n=91) | 0.0% (n=2) | 0.0% (n=107) |
| Validated | I543T | 0.0% (n=14) | 0.0% (n=91) | 0.0% (n=2) | 0.0% (n=110) |
| Validated | P553L | 0.0% (n=14) | 0.0% (n=91) | 0.0% (n=2) | 0.0% (n=110) |
| Validated | R561H | 0.0% (n=14) | 0.0% (n=91) | 0.0% (n=2) | 0.0% (n=110) |
| Validated | P574L | 0.0% (n=14) | 0.0% (n=91) | 0.0% (n=2) | 0.0% (n=109) |
| Validated | R622I | 0.0% (n=14) | 0.0% (n=91) | 0.0% (n=2) | 0.0% (n=108) |
| Validated | A675V | 0.0% (n=14) | 0.0% (n=91) | 0.0% (n=2) | 0.0% (n=109) |
| Candidate | P441L | 0.0% (n=14) | 0.0% (n=91) | 0.0% (n=2) | 0.0% (n=107) |
| Candidate | G449A | 0.0% (n=14) | 0.0% (n=91) | 0.0% (n=2) | 0.0% (n=107) |
| Candidate | C469F | 0.0% (n=14) | 0.0% (n=91) | 0.0% (n=2) | 0.0% (n=107) |
| Candidate | A481V | 0.0% (n=14) | 0.0% (n=91) | 0.0% (n=2) | 0.0% (n=107) |
| Candidate | R515K | 0.0% (n=14) | 0.0% (n=91) | 0.0% (n=2) | 0.0% (n=107) |
| Candidate | P527H | 0.0% (n=14) | 0.0% (n=91) | 0.0% (n=2) | 0.0% (n=109) |
| Candidate | N537I/D | 0.0% (n=14) | 0.0% (n=91) | 0.0% (n=2) | 0.0% (n=110) |
| Candidate | G538V | 0.0% (n=14) | 0.0% (n=91) | 0.0% (n=2) | 0.0% (n=110) |
| Candidate | V568G | 0.0% (n=14) | 0.0% (n=91) | 0.0% (n=2) | 0.0% (n=109) |
| ***Pfmdr1*** | | | | | |
| Validated | N86Y | 0.0% (n=13) | 4.5% (n=80) | 0.0% (n=62) | 2.4% (n=102) |
| Validated | Y184F | 80.2% (n=3) | 55.1% (n=81) | 55.6% (n=62) | 67.8% (n=101) |
| Validated | S1034C | 0.0% (n=3) | 0.0% (n=81) | 0.0% (n=62) | 0.0% (n=102) |
| Validated | N1042D | 0.0% (n=3) | 0.0% (n=81) | 0.0% (n=62) | 0.0% (n=102) |
| Validated | D1246Y | 0.0% (n=4) | 0.0% (n=80) | 0.0% (n=62) | 0.0% (n=102) |
| Haplotype | NFD | 0.0% (n=1) | 32.0% (n=25) | 100.0% (n=2) | 25.0% (n=32) |
| Haplotype | YFD | 100.0% (n=1) | 64.0% (n=25) | 0.0% (n=2) | 75.0% (n=32) |
| Haplotype | NYD | 0.0% (n=1) | 4.0% (n=25) | 0.0% (n=2) | 0.0% (n=32) |
| ***Pfcrt*** | | | | | |
| Validated | K76T | 0.0% (n=13) | 3.6% (n=85) | 1.9% (n=52) | 3.8% (n=101) |
| Candidate | C72S | 0.0% (n=13) | 0.0% (n=85) | 0.0% (n=52) | 0.0% (n=101) |
| Candidate | M74I | 0.0% (n=13) | 3.6% (n=85) | 1.9% (n=52) | 3.8% (n=101) |
| Candidate | N75E | 0.0% (n=13) | 2.4% (n=85) | 1.9% (n=52) | 2.9% (n=101) |
| Candidate | A220S | 0.0% (n=3) | 3.6% (n=85) | 0.0% (n=52) | 4.0% (n=98) |
| Candidate | Q271E | 0.0% (n=3) | 5.2% (n=85) | 2.3% (n=52) | 4.4% (n=98) |
| Candidate | N326S | 0.0% (n=3) | 0.0% (n=85) | 0.0% (n=52) | 0.0% (n=98) |
| Candidate | I356T | 0.0% (n=3) | 2.1% (n=85) | 2.6% (n=52) | 4.0% (n=100) |
| Candidate | R371I | 0.0% (n=3) | 5.1% (n=85) | 2.5% (n=52) | 5.3% (n=99) |
| Candidate | T93S | 0.0% (n=3) | 0.0% (n=85) | 0.0% (n=52) | 0.0% (n=101) |
| Candidate | H97Y | 0.0% (n=3) | 0.0% (n=85) | 0.0% (n=52) | 0.0% (n=101) |
| Candidate | F145I | 0.0% (n=3) | 0.1% (n=85) | 0.0% (n=52) | 0.0% (n=100) |
| Candidate | I218F | 0.0% (n=3) | 0.0% (n=85) | 0.0% (n=52) | 0.0% (n=99) |
| Candidate | C350R | 0.0% (n=3) | 0.0% (n=85) | 0.0% (n=52) | 0.0% (n=100) |
| Haplotype | CVIET | 0.0% (n=3) | 0.0% (n=41) | 0.0% (n=2) | 5.0% (n=60) |
| Haplotype | CVMNK | 100.0% (n=3) | 100.0% (n=41) | 100.0% (n=2) | 95.0% (n=60) |
| ***Pfdhfr*** | | | | | |
| Validated | N51I | 63.1% (n=14) | 88.4% (n=92) | 94.6% (n=52) | 89.0% (n=114) |
| Validated | C59R | 57.6% (n=14) | 93.9% (n=92) | 98.3% (n=52) | 92.5% (n=114) |
| Validated | S108N | 100.0% (n=14) | 95.1% (n=92) | 100.0% (n=52) | 95.4% (n=115) |
| Validated | I164L | 0.0% (n=14) | 0.0% (n=92) | 0.0% (n=52) | 0.0% (n=114) |
| Candidate | A16V | 0.0% (n=14) | 0.0% (n=92) | 0.0% (n=52) | 0.0% (n=115) |
| ***Pfdhps*** | | | | | |
| Vaildated | A437G | 66.6% (n=3) | 85.2% (n=91) | 95.6% (n=52) | 90.6% (n=115) |
| Validated | K540E | 0.0% (n=3) | 0.0% (n=91) | 0.0% (n=52) | 0.0% (n=114) |
| Candidate | S436A/F | 33.2% (n=3) | 63.4% (n=91) | 59.5% (n=52) | 57.2% (n=115) |
| Candidate | A581G | 0.0% (n=3) | 15.4% (n=91) | 0.0% (n=52) | 18.7% (n=113) |
| Candidate | A613S | 40.2% (n=3) | 27.8% (n=91) | 24.2% (n=52) | 31.6% (n=113) |
| Candidate | A613T | 0.0% (n=3) | 0.0% (n=91) | 0.0% (n=52) | 0.0% (n=113) |
| ***Pfdhfr + Pfdhps haplotypes**** | | | | | |
| Haplotype | IRN/AGKAA (quintouple) | 0.0% (n=2) | 19.0% (n=26) | 100.0% (n=2) | 15.0% (n=27) |
| Haplotype | IRN/VAGKAA (sextuple) | 0.0% (n=2) | 12.0% (n=26) | 0.0% (n=2) | 3.7% (n=27) |
| Haplotype | IRN/IAAKAA (triple) | 0.0% (n=2) | 3.8% (n=26) | 0.0% (n=2) | 11.0% (n=27) |
| Haplotype | IRN/SGKAA (quadruple) | 50.0% (n=2) | 38.0% (n=26) | 0.0% (n=2) | 41.0% (n=27) |
| Haplotype | NCS/SGKAA (quadruple) | 0.0% (n=2) | 3.8% (n=26) | 0.0% (n=2) | 0.0% (n=27) |
| Haplotype | IRN/VAGKGS (septuple) | 0.0% (n=2) | 7.7% (n=26) | 0.0% (n=2) | 22.0% (n=27) |
| %: weighted VAF across reads  (n=): number of samples contributing to site-level estimate  Site-level values represent the variant allele frequency, calculated as the proportion of mutant reads to total reads at each locus, averaged across all successfully sequenced samples. The percentage therefore reflects a read-based measure, while the number in parentheses indicates the number of samples with adequate sequencing coverage at that locus. | | | | | |
| **dhfr* haplotypes defined at codons: 51, 59, 108  *dhps* haplotypes defined at codons: 436, 437, 540, 581, 613  Wildtype: NCS/SAKAA | | | | | |
